# Iodinated Contrast-Induced Thyroid Storm With Concomitant Cardiac Tamponade: A Case Report

**DOI:** 10.7759/cureus.28001

**Published:** 2022-08-14

**Authors:** Tasnuva Amin, Christopher P Austin, Ndausung Udongwo, Kyle Wiseman, Amardeep S Parhar, Saira Chaughtai

**Affiliations:** 1 Internal Medicine, Jersey Shore University Medical Center, Neptune, USA; 2 Internal Medicine, St. George’s University School of Medicine, True Blue, GRD

**Keywords:** graves´disease, hyperthyroidism, contrast-induced thyroid disorders, jod-basedow, thyroid-storm

## Abstract

A thyroid storm is a rare but life-threatening condition caused by the exaggeration of the clinical manifestations of thyrotoxicosis. The symptoms of thyroid storms are non-specific in nature, making the Burch Wartofsky Point Scale (BWPS) a valuable resource in the diagnosis of thyroid storms. Though not part of the BWPS scoring criteria, literature reviews have shown an increasing number of reports of pericardial effusion secondary to thyrotoxicosis. Pericardial effusion is a well-known sequela of hypothyroidism that, in severe cases, can cause cardiac tamponade. With increasing reports, clinicians should be aware that pericardial effusions can develop secondary to both underactive and overactive thyroid states. We present a case of iodine contrast-induced Jod-Basedow in a 29-year-old with a prior history of Graves disease with concomitant pericardial effusion requiring thyroidectomy.

## Introduction

A thyroid storm is a rare but life-threatening condition of hyperthyroidism. It is triggered by either an acute event such as iodinated contrast administration, trauma, thyroid or non-thyroidal surgery or infection. One of the most common causes of a thyroid storm is the irregular usage or discontinuation of antithyroid medications [1]. With the advancement in medicine over the years, there has been an increased use of several radiologic imaging techniques requiring contrast media for diagnosis and management [2]. Although patients with functionally active thyroids could withstand the iodine load that comes with these imaging modalities, those with prior thyroid dysfunction particularly are at a greater risk of an abrupt hyper or hypo-thyroid dysfunction [2]. The prevalence of iodinated contrast-induced thyroid dysfunction ranges from 1-15% [2]. We present a case of iodine contrast-induced Jod-Basedow in a 29-year-old with a prior history of Graves' disease with concomitant pericardial effusion requiring thyroidectomy.

## Case presentation

A 29-year-old man with a past medical history significant for Graves' disease diagnosed one year prior, which was poorly managed with methimazole due to noncompliance, was admitted to our hospital with complaints of chest pain for one day. About four months prior, he was admitted for a syncopal episode leading to a motor vehicle accident. During that admission, he was found to have acute pericarditis with an initial concern for traumatic hemopericardium. However, repeat cardiac imaging showed only a small effusion with a minimal hemorrhagic component and the patient was successfully treated with colchicine.

**Figure 1:**
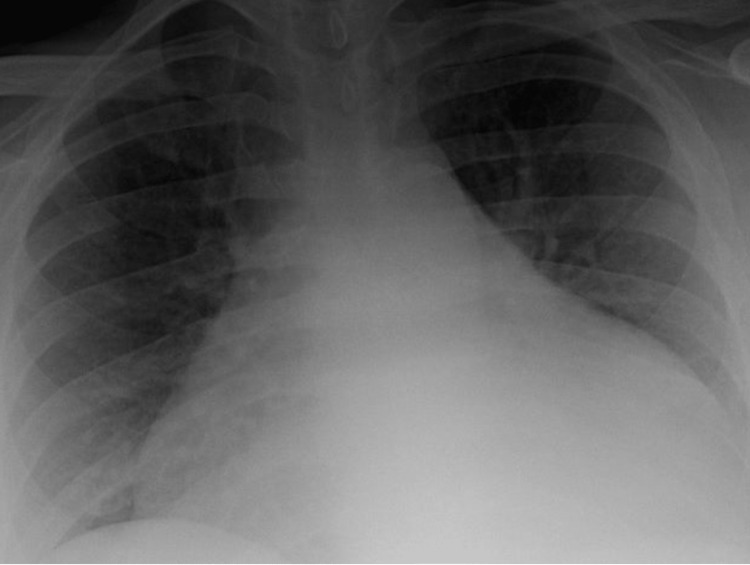
Enlarged cardiac silhouette with clear lung fields bilaterally

**Figure 2:**
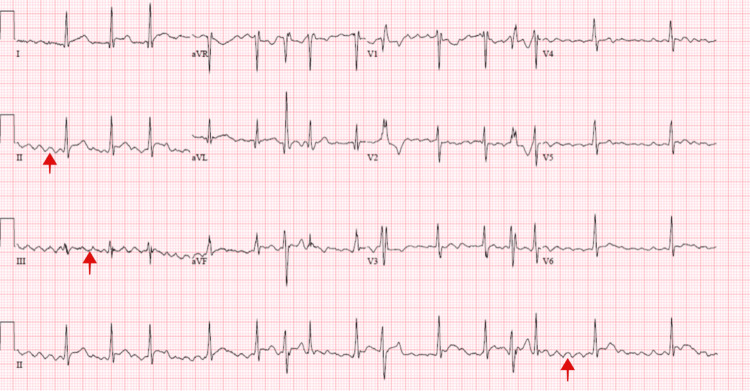
Electrocardiogram showing an irregular rhythm with aberrantly conducted complexes

**Figure 3:**
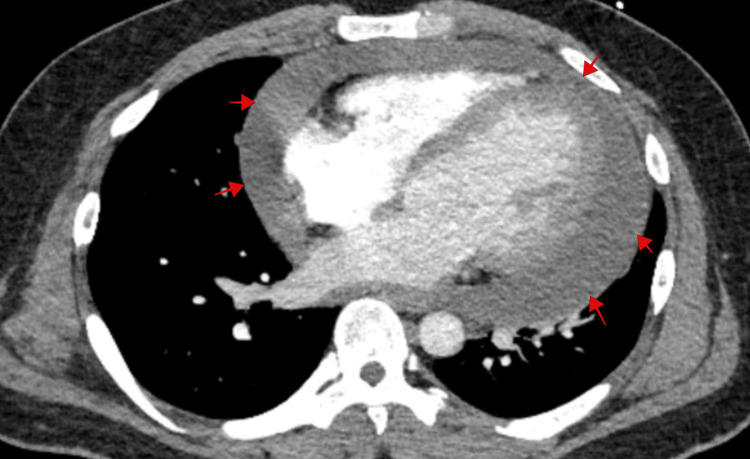
Computed tomography angiogram of the chest showing large pericardial effusion (red arrows) with borderline pericardial thickening and the presence of mild concavity of the left atrium

**Figure 4:**
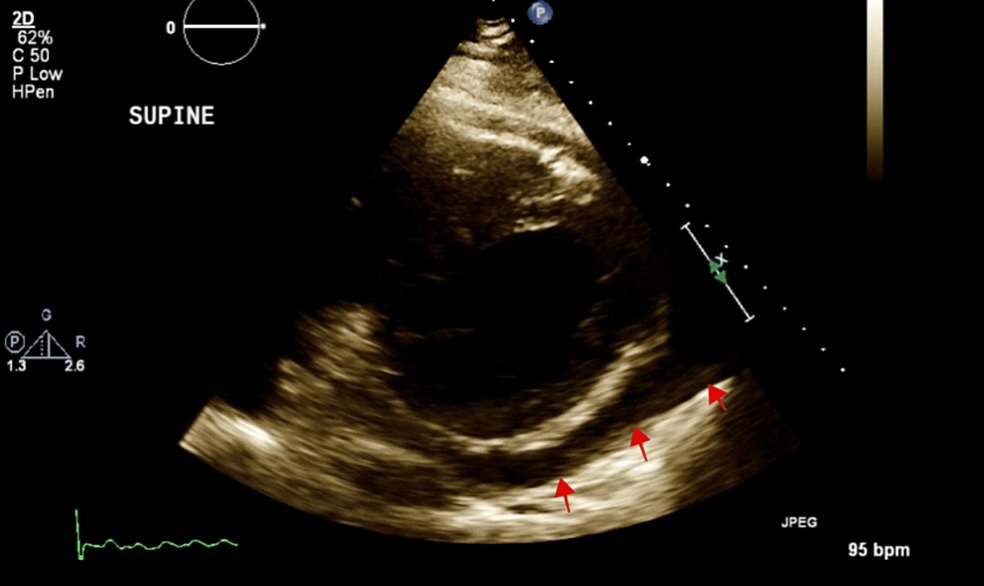
A 2D-Echocardiography showing moderate pericardial effusion (red arrows).

**Figure 5:**
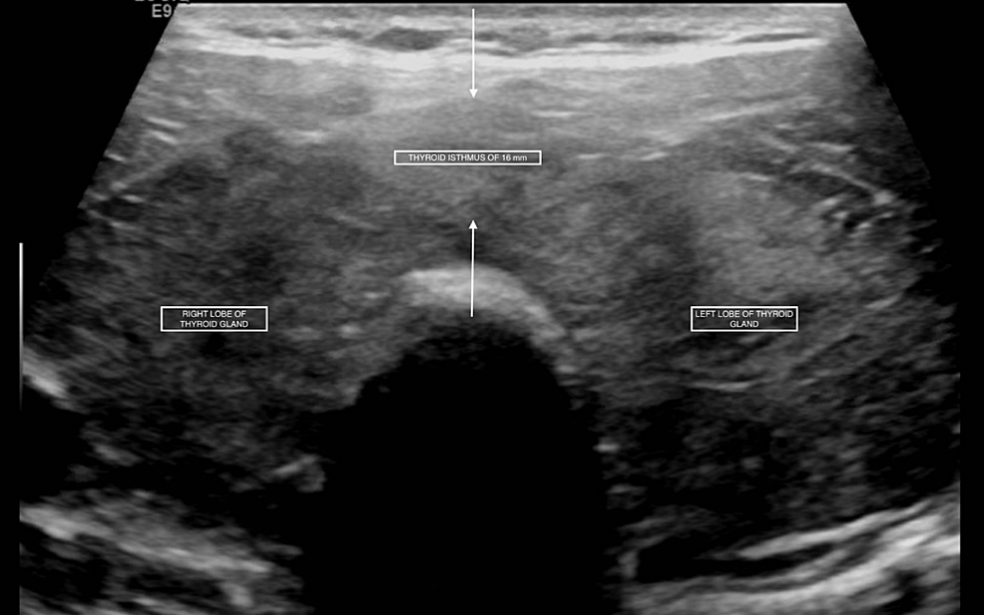
An enlarged heterogeneous hypervascular thyroid gland (right & left thyroid lobes) without discrete nodules

The patient returned with substernal, pleuritic chest pain. The pain was non-radiating, aggravated with deep inhalation and supine positioning with no known alleviating factors. It was associated with palpitations, diarrhoea and a globus sensation in the neck. The patient endorsed a two-year history of hyperthyroidism diagnosed during an evaluation for new-onset atrial fibrillation and underwent radiofrequency ablation. He was then discharged on methimazole. Labs on presentation showed normal troponin, suppressed thyroid-stimulating hormone, elevated free T4, elevated T3 levels and no thyroid peroxidase antibodies (Table 1). Initial chest radiography showed a markedly enlarged cardiac silhouette with clear lung fields (Figure 1). Electrocardiography showed atrial fibrillation (Figure 2). Computed tomography (CT) angiogram of the chest with IV iodinated contrast redemonstrated the large pericardial effusion and borderline pericardial thickening (Figure 3). Transthoracic echocardiography revealed a significant pericardial effusion with tamponade physiology (Figure 4). A thyroid ultrasound revealed an enlarged heterogeneous hypervascular thyroid gland without discrete nodules (Figure 5).

**Table 1:** The patient's laboratory findings at presentation

Laboratory Study	Result	References
Hemoglobin (g/dL)	11.7 (g/dL)	12.0-16.0 (g/dL)
White blood cells (10*3u/L)	9.8 (10*3u/L)	4.5-11.0 (10*3u/L)
Glucose (mg/dL)	106 (mg/dL)	70-99 (mg/dL)
Creatinine (mg/dL)	1.07 (mg/dL)	0.61-1.24 (mg/dL)
Sodium (mmol/L)	131 (mmol/L)	136-146 (mmol/L)
Calcium (mg/dL)	9.3 (mg/dL)	8.4-10.2 (mg/dL)
Potassium (mmol/L)	4.5 (mmol/L)	3.5-5.0 (mmol/L)
Phosphorus (mmol/L)	4.0 (mmol/L)	3-4.5 (mmol/L)
Magnesium (mg/dL)	2.0 (mg/dL)	1.3-2.5 (mg/dL)
Thyroid-stimulating hormone (TSH) (uIU/mL)	<0.010 (ulU/mL)	0.3-4.5 (uIU/mL)
T3	13.6 (pg/mL)	2.3-4.2 (pg/mL)
Free T4	3.19 (ng/mL)	0.5-1.26 (ng/mL)
Troponin	0.01 (ng/mL)	<0.04 (ng/mL)
Aspartate aminotransferase	36 (U/L)	10-42 (U/L)
Alanine aminotransfarase	56 (U/L)	10-60 (U/L)

Anti-thyroid medication treatment with 5 mg methimazole three times per day was initiated. He underwent CT-guided insertion of a pericardial drain for large symptomatic pericardial effusion which drained about 1500 cc of serosanguineous fluid. He developed uncontrolled atrial fibrillation with a rapid ventricular response and was placed on Diltiazem infusion, which failed to control the arrhythmia. He was thus transferred to the intensive care unit due to hemodynamic instability Endocrinology consultation yielded a Burch-Wartofsky score of 45 (normal: <25), consistent with thyroid storm, thought to be partly due to the IV iodinated contrast received early in the hospital stay. He was started on 100 mg intravenous hydrocortisone every eight hours, with methimazole being changed to 200 mg propylthiouracil every 6 hours. He was also started on one packet of cholestyramine every six hours and 100 mg oral metoprolol-succinate twice daily. An ultrasound of the thyroid revealed an enlarged, heterogeneous hypervascular thyroid gland without discrete nodules, consistent with “Graves' disease”. On day three of hospitalization, the heart rate showed marked improvement with a significant decrease in Free T4 to 1.54 ng/mL. The patient then underwent a total thyroidectomy. On day 10, he was discharged in stable condition and advised to follow up as an outpatient. Three months status post thyroidectomy the patient has had no recurrence of pericardial effusion or any other associated complications. 

## Discussion

With an incidence of 3%-37%, pericardial effusion is a well-known sequela of hypothyroidism that, in severe cases, can develop into cardiac tamponade [3-5]. Pericardial effusion secondary to hyperthyroidism is a rarely reported phenomenon; however, clinicians should be aware that these events can occur in both the underactive and overactive thyroid states [6-12]. The pathophysiology of thyrotoxic pericardial effusion is not fully understood but some researchers theorize that it may be similar to that of pretibial myxedema, with a serous effusion secondary to the transudation of albumin and decreased lymphatic clearance of interstitial fluid proteins [7,12]. It should be noted that effusion characteristics can vary and evaluation of fluid can help identify various etiologies [6].

Our patient, with a known history of Grave’s disease, presented to our hospital with atrial fibrillation, tachycardia with a rate into the 130s, moderate gastrointestinal-hepatic dysfunction, and pleuritic chest pain for the past day. This thyrotoxic state at presentation progressed to a fulminant thyroid storm after the administration of iodine-based contrast for a CT angiogram of the chest on admission.

This phenomenon, known as Jod-Basedow syndrome, is a rare yet well-reported cause of thyrotoxicosis following the administration of exogenous iodine [13-14]. Iodine is an essential mineral that is necessary for proper thyroid function and hormone production, with any fluctuation in levels adversely affecting how well the thyroid functions [15-16]. The Wolff-Chaikoff effect theorizes that as iodine rises, the synthesis of thyroid hormone will decrease secondary to an autoregulatory inhibition of iodine transport [16]. However, some patients are able to depart from this physiologic negative feedback loop and, in turn, progress into a thyrotoxic state [15]. Relevant to our patient, pre-existing thyroid disease is recognized as a risk factor in the development of iodine-induced hyperthyroidism [15]. It is imperative that precautions are taken when administering iodine in patients with preexisting thyroid pathologies. Initially, it was believed that our patient's preexisting hyperthyroidism was well managed with methimazole; this, however, was not the case and predisposed him to worsening thyroid function.

As our patient's thyrotoxic state progressed, so did his pericardial effusion. The patient quickly developed tamponade symptomatology. He became hemodynamically unstable with muffled heart sounds and jugular venous distention, prompting a therapeutic pericardiocentesis. The patient tolerated the procedure well, despite his ongoing atrial fibrillation.

After failed stabilization on diltiazem drip, the patient was worked up for a thyroid storm after receiving a Burch-Wartofsky Point Scale (BWPS) of 45. Due to thyroid storm symptoms being non-specific in nature, the BWPS has become a valuable resource in diagnosis for clinicians. The BWPS considers various precipitating factors from multiple organ systems, including but not limited to tachycardia, atrial fibrillation and gastro-hepatic dysfunction [17]. Once the diagnosis was confirmed, the patient was started on intravenous hydrocortisone, cholestyramine, and an oral beta-blocker. Methimazole was changed to propylthiouracil. A thyroid storm has an in-hospital mortality of 10.1%, so it was imperative for us to start treatment immediately in this patient [18]. This intervention was tolerated well by the patient and was very efficacious in bridging our patient for surgical thyroidectomy.

## Conclusions

In conclusion, thyroid storms, although a rare endocrine emergency, can have fatal outcomes characterized by the dysfunction of multiple organ systems. The occurrence of a thyroid storm in patients with a pre-existing and uncontrolled hyperthyroid state after iodinated contrast exposure is rare. In our patient, the initial presentation of pericardial effusion and subsequent occurrence of the Jod-Basedow phenomenon are both uncommon manifestations of a very common thyroid disorder. Pericardial effusions have long been associated with hypothyroidism. However, clinicians should be aware of the increasing number of reported pericardial effusions secondary to hyperthyroidism. Timely recognition of these atypical presentations and implementation of life-saving treatment is necessary for positive patient outcomes.
